# Early Diagnosis of Neutropenic Enterocolitis by Bedside Ultrasound in Hematological Malignancies: A Prospective Study

**DOI:** 10.3390/jcm10184277

**Published:** 2021-09-21

**Authors:** Edoardo Benedetti, Benedetto Bruno, Francesca Martini, Riccardo Morganti, Emilia Bramanti, Francesco Caracciolo, Sara Galimberti, Piero Lippolis, Emanuele Neri, Chiara Arena, Francesca Cerri, Vittorio Ricchiuto, Matteo Pelosini, Enrico Orciuolo, Mario Petrini

**Affiliations:** 1 Bone Marrow Transplant Unit, Department of Clinical and Experimental Medicine, UO Hematology, Azienda Ospedaliero-Universitaria Pisana, University of Pisa, 56126 Pisa, Italy; m_88_f@libero.it (F.M.); francaracciolo@gmail.com (F.C.); sara.galimberti@med.unipi.it (S.G.); mpelo78@hotmail.com (M.P.); e.orciuolo@alumni.sssup.it (E.O.); mario.petrini@med.unipi.it (M.P.); 2 Italian School of Basic and Emergency Ultrasound (SIUMB), 56100 Pisa, Italy; 3 Department of Molecular Biotechnology and Health Sciences, University of Turin, 10124 Torino, Italy; benedetto.bruno@unito.it; 4 Section of Statistics, Azienda Ospedaliero-Universitaria Pisana, 56126 Pisa, Italy; r.morganti@ao-pisa.toscana.it; 5 Institute of Chemistry of Organometallic Compounds (ICCOM-CNR), 56124 Pisa, Italy; bramanti@pi.iccom.cnr.it; 6 Department of General and Peritoneal Surgery, Azienda Ospedaliero-Universitaria Pisana, 56126 Pisa, Italy; pierolippolis@gmail.com; 7 Department of Radiodiagnostic 3, Azienda Ospedaliero-Universitaria Pisana, 56126 Pisa, Italy; emanuele.neri@med.unipi.it (E.N.); chiaraarena@yahoo.it (C.A.); francerri@gmail.com (F.C.); 8 Department of Health Technologies, ESTAR, Azienda Ospedaliero-Universitaria Pisana, 56126 Pisa, Italy; ing.vricchiuto@libero.it

**Keywords:** neutropenic enterocolitis, ultrasound sonography, intestinal infections

## Abstract

(1) Background: Neutropenic enterocolitis (NEC) is a life-threatening complication following chemotherapy with high mortality rates. Early diagnosis is crucial to improve outcomes. We designed a large prospective study employing bedside ultrasonography (US) as a novel approach to allow early diagnosis and prompt treatment to reduce mortality. (2) Methods: NEC was defined as US or computed tomography (CT)-proven bowel wall thickness ≥ 4 mm at the onset of at least one of the following symptoms: fever and/or abdominal pain and/or diarrhea during neutropenia. From 2007 to 2018, 1754 consecutive patients underwent baseline bedside US that was invariably repeated within 12 h from the onset of symptom(s) suggestive of NEC. (3) Results: Overall, 117 episodes of NEC were observed, and overall mortality was 9.4%. Bowel wall thickening was invariably absent in the negative control group. Abdominal pain associated with one or more symptoms correlated with the highest relative risk (17.33), sensitivity (89.7%), specificity (100%), and accuracy (96.2%) for diagnosis. The combination of abdominal pain and fever at onset significantly correlated with worse survival (*p* < 0.0001, OR 13.85). BWT (*p* = 0.046), type of therapy (*p* = 0.049) and blood culture positivity (*p* = 0.003) correlated with worse survival. (4) Conclusions: Bedside ultrasound is a non-invasive and radiation free imaging technique for early diagnosis of NEC and its prompt treatment significantly reduced mortality.

## 1. Introduction

*Neutropenic enterocolitis* (NEC) is a life-threatening clinical syndrome [1,2,3], characterized by fever, abdominal pain, and diarrhea during neutropenia. It is primarily observed in patients with hematological malignancies [4,5], but it has also been described in solid tumors and AIDS [2,6,7]. It was initially reported in pediatric patients undergoing treatment for leukemia or lymphoma and, then, reported in adults [6,7,8]. The incidence has been primarily evaluated in retrospective studies and ranged from 0.8% to 26% [3,4] with high mortality rates [2,8,9,10,11]. Early diagnosis and prompt treatment appear essential for survival [1,2,3,12,13,14]. Diagnostic criteria have been reviewed [4,5,15]. Bowel wall thickening (BWT), determined either by computed tomography (CT) or ultrasonography (US), has been proposed as a major diagnostic criterion [8,15,16]. Here we present a novel prospective study where non-invasive and cost-effective bedside ultrasound monitoring allowed early diagnosis and guided prompt medical treatment [3,11,17] resulting in significantly reduced mortality.

## 2. Materials and Methods

### 2.1. Patients and Study Design

Between March 2007 and January 2018, 1754 consecutive adult patients admitted to the Division of Hematology and to the Bone Marrow Transplant Unit of the University of Pisa, Italy, experiencing chemotherapy-related neutropenia were prospectively enrolled in our study. No up-front patient selection was performed, according to Gorschluter metanalysis [3]. The endpoint of the study was to prospectively verify the hypothesis that bedside ultrasonography could detect early signs of NEC leading to prompt medical treatment, eventually, reducing mortality. We considered statistically significant a reduction of NEC-related mortality from 30% [3] to 10% in standard conditions (α error 5% and power 80%). The patient population included all patients undergoing intensive chemotherapy or a transplant procedure, either autologous [18] or allogeneic [19,20], for the treatment of their hematological malignancies. In patients undergoing allogeneic stem cell transplantation acute GVDH and CMV colitis were excluded with bed side rectal biopsy [21,22]. Each admission was considered an “observation”, and each “observational period” started on the date of admission and ended on the date of discharge. A single patient could experience multiple “events” of NEC if diagnosed during different observational periods. All the other patients who experienced chemotherapy-related neutropenia but did not experience an NEC episode during the entire observational period were considered as the control group (N = 1646 patients). All patients provided written informed consent and the study was approved by the Institutional Review Board (IRB File 3636) according to the Declaration of Helsinki (identifier NCT04813679, https://clinicaltrials.gov/ct2/show/NCT04813679, accessed on 24 March 2021).

### 2.2. Definition of Neutropenic Enterocolitis

NEC was defined as bowel wall thickness ≥ 4 mm at the onset of at least one of the following symptoms: fever (axillary temperature ≥ 38.0 °C, F) and/or abdominal pain (AP) and/or diarrhea (more than three fluid stools/24 h, D) during neutropenia [3,5,15], which was defined as absolute neutrophil count (ANC) < 0.5 × 10^9^/L. Abdominal pain was evaluated using a Visual Analogous Scale Pain Score, ranging from 0 to 10 [5]. Resolution of NEC was defined as a complete disappearance of symptoms combined with “restitutio ad integrum” of all bowel segments involved at diagnosis by bedside ultrasound.

### 2.3. Antimicrobial Prophylaxis

From the start of the study until December 2013, all patients received levofloxacin 500 mg/day, fluconazole 400 mg/day, and aciclovir 400 mg twice a day until neutrophil recovery. In January 2014, levofloxacin and fluconazole prophylaxes were discontinued and patients with leukemia or undergoing allogeneic transplantation received posaconazole prophylaxis [23].

### 2.4. Microbiological Evaluation

**Figure 1:**
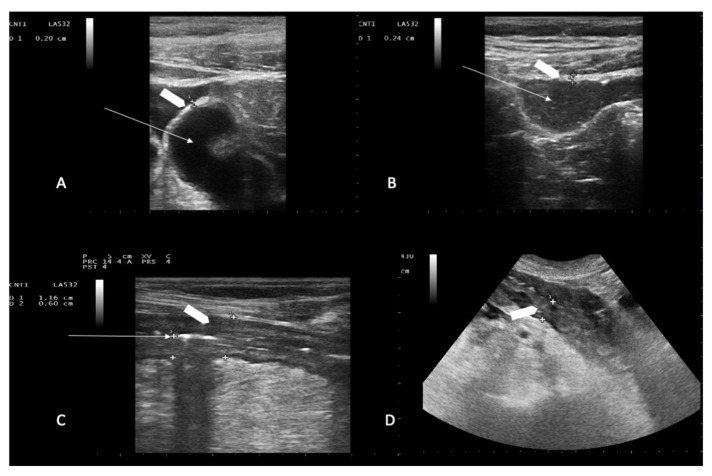
Representative ultrasound imaging of ileum loops. Chemotherapy-induced diarrhea: ileum loops filled with liquid (panel **A**) and mixed solid/liquid material (panel **B**). White arrows indicate the lumen; white arrowhead indicates normal bowel wall thickness (2.0 mm in panel **A**, and 2.4 mm in panel **B**). Neutropenic enterocolitis involving the terminal ileum loop and descending colon: white arrow in **C** shows compressed lumen (panel **C**); white arrowheads show bowel wall thickness (6.0 mm in panel **C**, and 13 mm in panel **D**). Bowel wall layers are recognizable in (panel **C**) while boundaries are poorly defined in (panel **D**).

Blood cultures were part of routine fever workup for all febrile episodes as per institutional policy as previously described [16]. Routine stool cultures were performed at each episode of diarrhea to rule out *Clostridium Difficile* colitis [5], or other bacterial or fungal infections. PCR analysis was carried out to rule out viral infections (herpes viruses, adenovirus, EBV, CMV, rotavirus, norovirus enterovirus, and astrovirus). Stool cultures were repeated if diarrhea persisted or worsened. Moreover, diarrhea was considered chemotherapy-induced [24] (Figure 1A,B) when no pathogen was isolated from stool cultures, and neutropenic fever was considered of unknown origin if no infection was microbiologically documented using extensive microbiological evaluation [5,11,18].

### 2.5. Ultrasonographic Examination

Each patient enrolled in the study underwent a baseline abdominal and intestinal B-mode US at the beginning of each observational period, as soon as admitted on the ward, before receiving any chemotherapy [25,26]. All patients were clinically monitored during their entire hospital stay (observational period). Ultrasound was performed with an Esaote My Lab 25 ultrasonographer equipped with a 3.5–5.0-MHz convex probe and a 7.5-MHz linear transducer without any preparation, at the onset of symptom/s. The entire gastrointestinal tract was submitted to a gray-scale US examination as previously described [25,27,28,29]. Bowel wall layers including superficial mucosal interface, deep mucosa, submucosa, muscolaris propria and serosa [30,31], degree of dilation [32] and motility [27]; presence of haustra or dehaustration and presence/absence of free abdominal fluid in all four quadrants and/or abdominal organ pathologies (such as Cholecystitis or hepatolienal candidiasis) [3,5] were assessed during each imaging study [27,29,33]. Bowel wall was defined as thickened if ≥4 mm in at least 3-cm-long segments in transvers scans [5,16,34,35] (Figure 1C,D), and bowel content was defined as gas, foodstuff or feces, mixtures of the two, or fluid filled [36]. Asymptomatic patients received another bed side US to assess the gastrointestinal tract after five days of neutropenia. If they did not experience an NEC episode during the entire observational period, they were considered controls. Follow-up ultrasound was invariably performed 6–12 h within the onset of either one or more symptoms and repeated if clinical conditions worsened or at onset of new symptoms. Patients diagnosed with NEC were considered our study group. Ultrasound studies were performed either on weekdays or during the weekend as clinically indicated. In all patients, bed-side US was performed by a hematologist member and teacher at the Italian School for Basic and Emergency Ultrasound (SIUMB) at the University of Pisa, with expertise in GIUS ultrasound.

### 2.6. Treatment

After the ultrasonographic diagnosis of NEC, blood cultures were obtained in febrile patients, and a conservative approach with broad-spectrum antibiotics covering both Gram-positive and -negative pathogens, anaerobes, and fungi, was immediately started regardless of symptoms. Treatment included meropenem, vancomicin, liposomal ampho-B; dosage was adjusted to renal function and caspofungin was used in patients with renal impairment and electrolyte imbalance. In sepsis/septic shock, IgM-enriched immunoglobulins were infused over three days [37,38]. Treatment was modified if infections were documented according to sensitivity tests. Patients also received total parenteral nutrition, G-CSF, fluid resuscitation, transfusions of packed red blood cells, platelet and fresh frozen plasma as needed [3,5]. NEC-related mortality was defined according to what was previously published [1,2,3,5,11,13,18,36].

### 2.7. Statistical Methods

Patients diagnosed with NEC were considered the study group. All the other patients who did not experience NEC episodes were considered as the control group. Each episode of NEC was an event. Categorical data were described by frequency (absolute and relative), whereas continuous data by median (IQR) or mean (sd). The comparison between qualitative variables was performed by Chi-square test and *z*-test for two proportions, while continuous data were analyzed by *t*-test (two-tailed). Multivariate analysis was carried out using a stepwise binary logistic regression. Sensitivity, specificity, and accuracy of symptoms were calculated. Finally, the correlation between year of diagnosis and ratio number of NEC death/number of NEC diagnoses was performed by Spearman correlation analysis. Significance was fixed at 0.05. All analyses, descriptive and inferential, were performed by SPSS v.27 technology.

## 3. Results

**Figure 2:**
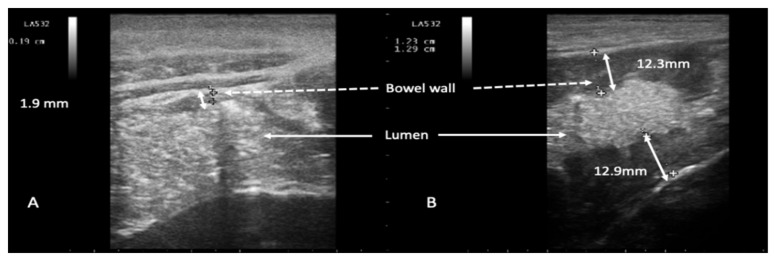
Representative ultrasound imaging of an episode of neutropenic enterocolitis evolved from chemotherapy-induced diarrhea. Chemotherapy-induced diarrhea: mixed solid and liquid content consistent with diarrheal feces in the lumen (panel **A**). Neutropenic enterocolitis evolved from chemotherapy-induced diarrhea (panel **B**): appearance of abnormal bowel wall thickness. White arrows indicate the colon lumen (panels **A**,**B**); dotted white arrows indicate bowel wall; double white arrows indicate bowel wall thickness (normal, 1.9 mm, in panel **A**, and abnormally thickened in panel **B**, 12.3 mm and 12.9 mm).

Overall, during the study period, we observed 117 episodes of NEC. One hundred and seventeen episodes of NEC occurred in 108 patients as 9 patients experienced two episodes of NEC, each during two different admissions. NEC developed after a median of 4 days of chemotherapy-related neutropenia (range 3–7 days). The overall incidence of NEC was 6.6% (117 NEC episodes/1754 patients). Two US examinations (median value, range 2–5) were performed for each episode. Mean BMI was 24.9 (range 17.58–35.64) in the NEC group and 24.5 (range 12.21–35.56) in the control group. Body mass index (BMI) did not impact the performance of the imaging technique (*p* = 0.504). Patient characteristics are reported in Table 1. Overall, 37 patients met the diagnostic criteria for NEC presenting with abrupt onset of abdominal pain associated with BWT (Figure 2) following chemotherapy-induced diarrhea (Figure 1A,B).

### 3.1. Presenting Symptoms and Intestinal Involvement

At diagnosis, fever was present in 67/117 (57%) episodes; in the 50/117 (43%) episodes without fever, presenting symptoms were abdominal pain and diarrhea in 38/50 (76%), abdominal pain only in 9/50 (18%), and diarrhea only in 3/50 (6%). Median BWT of the entire NEC group was 8.0 mm (range 5.9–30 mm). Overall, in 88 episodes, BWT was ≤10 mm and in 29 > 10 mm. Univariate and multivariate analysis evidenced that BWT > 10 mm was significantly associated with worse survival (*p* ≤ 0.0001, OR 10.79, and *p* = 0.046, respectively. Table 2).

Abnormal BWT was not observed in the control group. In NEC episodes in which NEC was resolved, BWT was 7.9 mm (median value, range 4.3–30 mm, sd 3.5). In NEC episodes in which NEC was the cause of death, BWT was 11.1 mm (median value, range 7–15 mm, sd 2.2) (*p* = 0.004). By ROC analysis, the best cutoff for survival was 9.25 mm (*p* = 0.0003, sensitivity 91%, specificity 77%). The localization of NEC either in the small bowel or in both the small bowel and the colon were significantly correlated with increased mortality in univariate analysis (*p* = 0,021, OR 4.58, and *p* = 0.009, OR 4.85, Table 2). Overall, in surviving patients, abdominal pain was the first symptom to resolve (24 h median time, range 20–29 h) from the start of the treatment, followed by fever and eventually diarrhea. The resolution of BWT took longer from 4 up to 9 days. The improvement of signs and symptoms was not associated with recovery from neutropenia that persisted for 6 days (median value, range 5 to 11) from the start of the treatment. Sensitivity, specificity, and accuracy of symptom combinations for the diagnosis of NEC are reported in Table 3. Abdominal pain associated with other symptom/s is correlated with the highest relative risk (RR) (17.33), sensitivity (89.7%), specificity (100%) and accuracy (96.2%).

### 3.2. Microbiological Studies

Overall, positive blood or stool cultures, or both, were found in 46/117 (39.3%) of NEC episodes: in 35/117 (29.9%) only positive blood cultures, in 9/117 (7.7%) only positive stool cultures, and in 2/117 (1.7%) both. Univariate (*p* < 0.0001, OR 13.85) and multivariate analysis (*p* = 0.003) results showed that only positive blood cultures were significantly associated with worse survival (Table 2). Bacterial strain isolates from blood cultures were Gram+ (*Staphylococcus epidermidis*, *Enterococcus faecalis*, *Staphylococcus aureus*) (in 38.8% episodes), Gram− (*Escherichia coli*, *Klebsiella pneumoniae*) (in 27.7% episodes), both Gram+ and Gram− (in 22.2% episodes), *Candida* (*Albicans* and *Krusei*) (in 11.3% episodes).

### 3.3. Treatment

**Figure 3:**
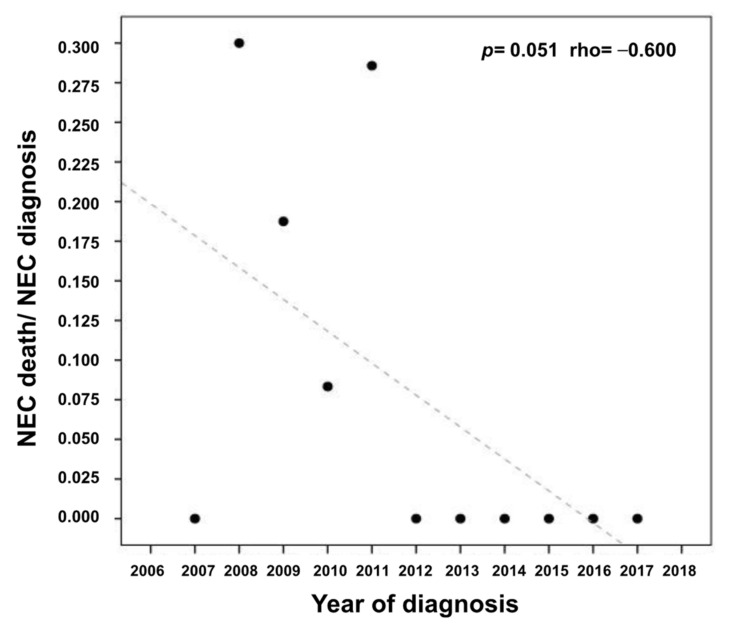
Correlation of mortality rate and incidence of neutropenic enterocolitis per year (*p* = 0.051, rho = −0.600).

Overall, in 111/117 (95%) NEC episodes, the patients received medical treatment. Six patients underwent surgery: 3 had small bowel resections, 2 right hemicolectomy, and 1 appendicectomy with resection of the last ileum loop extended to the cecum. Median time from the diagnosis to surgery was 9.5 h (range 5–12 h). Three out of 6 are alive and without any recurrence of NEC at a median follow-up of 7.5 years (range 4–8.5 years). Both univariate (*p* = 0.005, OR 12.87) and multivariate analysis (*p* = 0.049) results show that conservative medical treatment, as compared to surgery, is associated with a statistically significant improved survival (Table 2). Antimicrobial prophylaxis was discontinued in 2014 except for the introduction of posaconazole prophylaxis for acute myeloid leukemia in first induction [23]. No statistically significant impact (*p* = 0.780) was found either on the yearly incidence of NEC or on mortality before 2014 (mean of 10 episodes/year, N = 8 years, sd = 4.598), and after 2014 (9.25/year, N = 4 years, sd = 3.403). Mortality continued to decrease remarkably over time (*p* = 0.051, rho = −0.600) (Figure 3).

### 3.4. Mortality

Overall, 11 patients died from septic shock because of NEC with a mortality rate of 9.4% (11 patients out of 117 NEC episodes). This mortality rate is considered statistically significant (*p* = 0.003) in respect to 30% NEC-related mortality reported in the literature [3]. Median time from diagnosis to death was 26 h (range 10.5–72). Mortality rates were 22.2% (2/9) in patients who experienced 2 NEC episodes and 8% (9/99) in patients who experienced only one NEC episode, statistically not significant (*p* = 0.503). Univariate and multivariate analysis results showed that mortality was significantly higher in patients who received surgery with respect to medical treatment (Table 2). There was no difference between newly diagnosed patients who were febrile (8/67, 12%) or afebrile (3/50, 6%) (*p* = 0.413). No increased mortality (*p* = 0.125) was observed in patients who developed NEC following chemotherapy-induced diarrhea. Mortality down trended throughout the study period (*p* = 0.051 rho = −0.600) (Figure 3).

## 4. Discussion

NEC is a life-threatening abdominal infection [5]. Though likely multifactorial, its pathogenesis is primarily due to severe chemotherapy-induced mucosal damage, with infiltration of the underlying layers by luminal pathogens, that may easily result in uncontrolled sepsis in immune-compromised hosts [15,39,40,41]. Its reported incidence greatly varies from 0.8% to 46%, partly due to the retrospective nature and limited patient series of most studies, partly due to different definitions and diagnostic criteria [4,15,39,40,41,42,43,44]. In our prospective study, its incidence was 6.6%. 

It is widely assumed that NEC is a clinical diagnosis. Patient clinical conditions and thrombocytopenia often preclude tissue biopsies given the risk of bleeding and perforation. Thus, clinical symptoms combined with bowel wall thickening are considered highly suggestive diagnostic criteria [3,20,45]. Importantly, current non-invasive high-resolution ultrasound techniques allow detailed differential diagnosis including NEC [5,15,18,25], adding diagnostic sensitivity and avoiding radiation exposure as compared to CT [25]. Overall, ultrasound has been increasingly employed to evaluate the gastrointestinal tract of patients with hematological malignancies [20,25,26,29,34,35,45]. In NEC, bed side US could become a valid tool to be combined with patient clinical assessment and lead to early diagnosis, which is crucial for outcomes.

In this study, we found that the most frequent presenting symptom is abdominal pain, with/without diarrhea, which was invariably associated with an increased bowel wall thickness detected by bed-side ultrasound. Abdominal pain, combined with any of the other symptoms, correlated with the highest RR (17.33) for diagnosis of NEC and was also associated with higher diagnostic sensitivity, specificity, and accuracy (Table 3). Fever and diarrhea associated with other symptom/s had a RR for NEC diagnosis of 8.28 and 13.66, respectively. Fever has been described as a major diagnostic criterion by Gorschluter et al. [5] whereas as a minor one by Sachak et al. [15] and by Nesher et al. [4]. Of note, 43% of our patients were *afebrile* at diagnosis. This might be explained by the fact that ultrasound was promptly carried out also at the onset of a single symptom among abdominal pain, diarrhea, or fever. Bed-side ultrasound may detect the early phases of enterocolitis where the edema of the damaged mucosa, responsible for the BWT, determines abdominal pain. Fever further delayed by neutropenia and the paucity of inflammatory cells may become a late onset symptom, expression of the invasion of the bowel wall layers by infectious pathogens and incumbent sepsis [39], suggesting a multi-step pathogenesis [18]. Moreover, fever was never present as a single clinical symptom in our patient series. Importantly, regardless of the symptom at onset of NEC episodes, BWT was invariably present in the patients diagnosed with NEC whereas it was absent in the control group. Cartoni et al. reported that the wall thickness had prognostic relevance [36]. The mortality rate of patients with a wall thickness exceeding 10 mm was 27.5% in our cohort. Cartoni et al. [36] reported a remarkable mortality rate of 60% in patients with BWT > 10 mm. This difference may be due to the study design (prospective vs. retrospective). Nevertheless, even though the mortality in our study is lower, a wall thickness > 10 mm remains significantly associated with higher mortality also in our univariate and multivariate analysis (*p* = 0.0001 and *p* = 0.049, respectively).

Our standard of treatment was the prompt institution of broad-spectrum antibiotic chemotherapy, including antibacterial and antifungal agents, given the high risk of rapid evolution to sepsis. Treatment was changed only in the light of sensitivity studies if pathogens were isolated [3,11,23,40,46]. Blood cultures were positive in 39% of the episodes of NEC, similar to what was previously reported [4]. Antimicrobial therapy was continued until complete resolution of symptoms, normalization of white cell counts, and full recovery of the gastrointestinal function. Only 6 patients, though neutropenic and thrombocytopenic, underwent surgery [47,48] and three survived. Interestingly, surgical specimens showed mucosal and submucosal edema and occasional hemorrhagic findings further indicating that bowel wall thickening is a key event and reflects NEC pathophysiology [5,18,44]. 

The association of fungal infections with NEC may be underestimated [49]. In a systematic review of cases reports, mortality rates were 19% and 81% in patients who received and did not receive antifungal treatment, respectively [50]. In another study, fungal isolates, including both *Candida* and *Aspergillus* species, were documented in 16% of episodes before death, whereas in up to 53% post-mortem [7]. In a metanalysis [3], pooled frequency of fungal NEC was 6.2%, and *Candida* spp. were isolated in 94% of episodes with a mortality of 81.8%. These findings promptly influenced us to invariably administer antifungal agents.

In our study, univariate analysis found that symptoms, intestinal localization, positive blood cultures and type of treatment (medical vs. surgical) were key factors associated with the outcome. Multivariate analysis indicated that BWT, type of therapy and blood culture positivity resulted significant with respect to mortality (*p* = 0.046, *p* = 0.049 and *p* = 0.003, respectively).

The high mortality of NEC (30–60%) is due to sepsis, uncontrolled bleeding or necrotizing perforation [3,11]. In our prospective study, we showed that ultrasound performed within 12 h from the onset of any of the warning symptoms and prompt medical treatment may dramatically reduce the mortality: 9.4% in the entire series, 7% in the cohort treated with antibiotics, and 50% in patients treated with surgery. The mortality rate in our study is considered statistically significant with respect to 30% NEC-related mortality reported in the literature [3] (*p* = 0.003). In our study, we also found that patients experiencing two episodes of NEC did not have higher mortality with respect to patients experiencing only one NEC episode (*p* = 0.503). Importantly, other clinical observations included that the rapid improvement/resolution of abdominal pain, rather than that of other symptoms, was a key factor for good prognosis, and that neutrophil recovery did not consistently affect outcome. Moreover, the discontinuation of chinolonic prophylaxis did not affect either incidence or clinical outcomes. Of note, mortality decreased over time since 2007, though incidence per year and treatment of *NEC* did not vary. This may indicate that the use of ultrasound as a bedside, non-invasive diagnostic tool over the study period has allowed early diagnosis, and a progressive increase of timely treatments significantly impacted cure rates. Importantly, patient BMI did not affect the reliability of the ultrasound evaluation of the intestinal tract as previously reported [25,26,28,51]. Moreover, US is a very cost-effective technique, given that each procedure costs 36 euros by the tariff plan of the Italian National Health System in the Region of Tuscany.

## 5. Conclusions

Bed-side ultrasound is a valid tool for the early diagnosis of NEC. This imaging technique is non-invasive, radiation-free, widely available, and relatively inexpensive. Moreover, watchful bed-side monitoring with repeat imaging studies is feasible. Abdominal pain appeared the most representative clinical symptom at onset and bowel wall thickness, type of treatment and blood culture positivity, associated with prognosis. Prompt broad-spectrum antimicrobial therapy reduces mortality. Multi-center trials are warranted to confirm our prospective experience.

## Figures and Tables

**Table 1:** Patient characteristics.

Characteristics	Frequency (%) or Median (IQR)
Gender	
Male	61 (52%)
Female	56 (48%)
BMI	Mean 24.4 (IQR 22.2–27.6)
Disease	
AML	34 (29%)
NHL	39 (33%)
HL	20 (17%)
MM	13 (11%)
Others	11 (10%)
Therapy	
Allogeneic Tx	12 (10%)
Autologous Tx	56 (48%)
Chemotherapy	49 (42%)
Positive cultures bacterial cultures	
Blood	34 (29%)
Stool	9 (7%)
Both	2 (2%)
Involved sites	
Small bowel	20 (17%)
Colon	70 (60%)
Both	27 (23%)
BWT	
≤10 mm	88 (75%)
>10 mm	29 (25%)

Abbreviations: BMI: body mass index; AML: acute myeloid leukemia; NHL: non-Hodgkin lymphoma; HL: Hodgkin’ lymphoma; MM: multiple myeloma; Tx: transplant; BWT: bowel wall thickness.

**Table 2:** Univariate analysis of the impact of clinical factors and BWT on outcome in *n* = 117 episodes of NEC. The results of multivariate analysis are reported in the note.

Factor		Alive	Dead	OR	*p*-Value
BWT	≤10 mm	85	3	10.791	<0.0001
	>10 mm	21	8		
Fever	No	26	0	-	0.063
	Yes	80	11		
Pain	No	6	0	-	0.418
	Yes	100	11		
Diarrhea	No	13	1	1.398	0.758
	Yes	93	10		
NEC after diarrhea	No	70	9	0.432	0.468
	Yes	36	2		
Fever + diarrhea	No	33	1	4.521	0.125
	Yes	73	10		
Fever + pain	No	31	0	-	0.036
	Yes	75	11		
Diarrhea + pain	No	19	1	2.184	0.459
	Yes	87	10		
Fever + diarrhea + pain	No	38	1	5.588	0.073
	Yes	68	10		
Small bowel localization	No	67	3	4.581	0.021
	Yes	39	8		
Colon localization	No	18	2	0.92	0.92
	Yes	88	9		
Small bowel + colon localization	No	85	5	4.857	0.009
	Yes	21	6		
Blood culture positivity	No	80	2	13.85	<0.0001
	Yes	26	9		
Coproculture positivity	No	97	11	-	0.314
	Yes	9	0		
Coproculture + blood culture positivity	No	104	11	-	0.646
	Yes	2	0		
Type of therapy	Medical	103	8	12.87	0.005
	Surgery	3	3		

Note: BWT, type of therapy and blood culture positivity resulted as significant after multivariate analysis by stepwise method (*p* = 0.046, *p* = 0.049 and *p* = 0.003, respectively).

**Table 3:** Sensitivity, specificity, and accuracy of symptom combinations for the diagnosis of NEC.

Presenting Symptom/s	NEC	RR	Sensitivity (%)	Specificity (%)	Accuracy (%)
Only fever	0	0.00 ^1^	0.0	91.3	57.2
Only diarrhea	1	0.08	0.9	85.2	53.7
Only pain	6	1.07	5.1	95.4	61.7
Fever and other symptom/s	91	8.28	77.8	99	91.1
Diarrhea and other symptom/s	102	13.66	87.2	99	94.6
Pain and other symptom/s	104	17.33	89.7	100	96.2

^1^ N. episodes of NEC with only fever observed. Abbreviations: NEC: Neutropenic enterocolitis; RR: relative risk.
